# Genetic structure and historical demography of *Schizothorax nukiangensis* (Cyprinidae) in continuous habitat

**DOI:** 10.1002/ece3.1413

**Published:** 2015-02-02

**Authors:** Weitao Chen, Kang Du, Shunping He

**Affiliations:** 1 The Key Laboratory of Aquatic Biodiversity and Conservation of Chinese Academy of Sciences, Institute of Hydrobiology, Chinese Academy of SciencesWuhan, Hubei, 430072, China; 2 University of Chinese Academy of SciencesBeijing, 100049, People′s Republic of China

**Keywords:** Expansion event, genetic structure, isolated by distance, Nujiang River, population bottleneck, *Schizothorax nukiangensis*

## Abstract

Geographic distance, different living habitats or Pleistocene climatic oscillations have frequently been found to shape population genetic structure in many species. The genetic structure of *Schizothorax nukiangensis*, a high altitude, valuable fish species, which is distributed throughout the Nujiang River, was investigated by mitochondrial DNA sequence analysis. The cytochrome c oxidase subunit I (*COI*), cytochrome b (*cytb*), and the mitochondrial control region (*MCR*) of *S. nukiangensis* were concatenated for examination of population structure and demographic history. The concatenated data set (2405 bp) implied a pronounced genetic population structure (overall *F*_ST_ = 0.149) and defined two population units. Strong differentiation was detected between the Sanjiangkou (SJK) population and other populations due to environmental heterogeneity, dispersal ability, and/or glacial cycles. Additional DNA sequencing of the nuclear RAG2 gene also examined significant differentiation between two units and between SJK and the upstream populations (U-unit). Recent expansion events suggest that *S. nukiangensis* may have undergone a rapid increase during warm interglacial periods. Surprisingly, *S. nukiangensis* appears to have undergone an obvious expansion during the last glaciations (LG) for cold hardiness and a sharp contraction from 1.5 ka to the present. However, two population units exhibited different reflections during the LG, which might be closely related to their living habitats and cold hardiness. A clear pattern of isolation by distance was detected in *S. nukiangensis* due to feeding habits, limited dispersal ability, and/or philopatry. It is vitally important that more attention be given to *S. nukiangensis* due to low genetic diversity, lack of gene flow, and recent population contraction.

## Introduction

**Figure 1:**
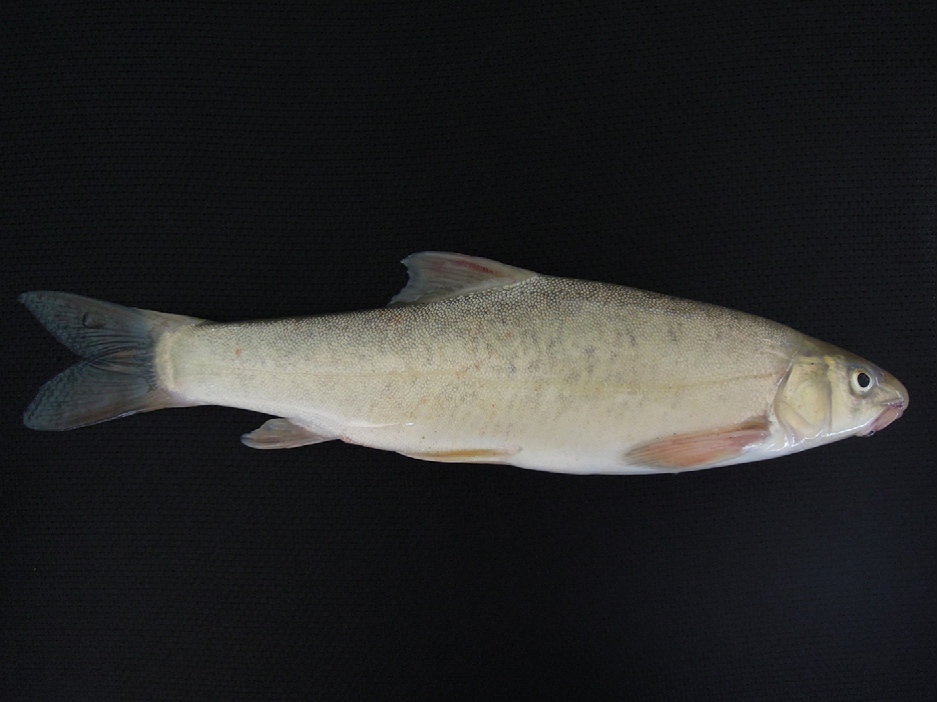
The study species, *Schizothorax nukiangensis* in the Nujiang River.

The Nujiang River is an important river in China that flows from north to south. This region has retained a rich biodiversity due to a changeable climate and unique geographic features. The Nujiang River is famous for its distinct fish fauna. In fact, a total of 77 species have been reported to reside in this river in China, many of which are endemic (Chu and Chen 1989; Chen 1998, 2013; Fu et al. 2012). However, few studies have been conducted examining fish in the Nujiang River, particularly the endemic species. Four schizothoracine fishes (*Schizothorax lissolabiatus*, *S. gongshanensis*, *S. yunnanensis paoshanensis,* and *S. nukiangensis*) have been documented in the Nujiang River system (Chu and Chen 1989; Chen and Cao 2000). *S. nukiangensis* (Cypriniformes: Cyprinidae) (Fig.1) is a plateau fish that is economically valuable. It is widely distributed in the Nujiang River and is specialized for high elevation, exhibiting a number of unique adaptations. However, the stock of *S. nukiangensis* has declined dramatically in recent years due to overfishing, mining, the construction of hydropower stations in some tributaries and environmental disruption. In addition, there are plans to build 13 dam cascades along the Nujiang River mainstream, which will exacerbate the decline of *S. nukiangensis* (Dudgeon 2011). Consequently, it is crucial that the current stock of *S. nukiangensis*, and its resources, be examined and protected.

Freshwater ecosystems all over the world have been heavily exploited and degraded by human activities, affecting both fish and fisheries; thus, it is vital that conservation efforts are undertaken quickly. Population genetic analysis is a widely used approach for assessing the genetic divergence in populations (Crandall et al. 1999) and for guiding conservation work. There is a growing body of population genetic structure studies primarily focusing on fragmented environments, while few have focused on continuous habitats. Therefore, in many species, we often know less about the patterns of genetic differentiation in continuous habitats (Cabe et al. 2007). However, understanding of patterns of genetic architecture in continuous habitats is important for determination of how these patterns are altered by fragmentation (Cabe et al. 2007). Therefore, it is critical that more population analyses are conducted in continuous environments. As *S. nukiangensis* is distributed throughout the Nujiang River, it is an ideal candidate for the study of population genetics in a continuous model.

Pleistocene climatic oscillations played an important role in the contemporary diversity in many species and communities (Hewitt 2000, 2004). Glacial cycles during the Quaternary Period resulted in the periodic expansions and contractions of population sizes and distribution ranges of species. In this study, the effects of climatic oscillations on the historical demography of *S. nukiangensis* were examined. Limited dispersal capacity can cause small-scale genetic differentiation in populations. Because of feeding habits of preying phytoplankton attached to the stone and hypognathous mouth of *S. nukiangensis* (Chu and Chen 1989; Chen 1998; Chen and Cao 2000), we hypothesize that *S. nukiangensis* has limited dispersal capacity and may fit the isolation by distance (IBD) model (Wright 1943). IBD, in the context of population genetics, is the process by which a genetic structure is generated via geographically restricted gene flow due to the fact that random genetic drift is occurring locally (Hardy and Vekemans 1999).

In this study, three mitochondrial DNA sequences (the cytochrome c oxidase submit I (*COI*), cytochrome b gene (*cytb*), and control region (*MCR*)) were employed as a mitochondrial concatenated data set (MCD) to evaluate genetic diversity, the population genetic structure, and population demographic history of *S. nukiangensis*. Furthermore, whether IBD plays a role in shaping the structure of *S. nukiangensis* populations was investigated and whether the height of water acts as barrier to gene flow among *S. nukiangensis* populations was examined. This study seeks to develop meaningful recommendations for conservation policies and the preservation of *S. nukiangensis*.

## Materials and Methods

### Samples and laboratory analyses

**Figure 2:**
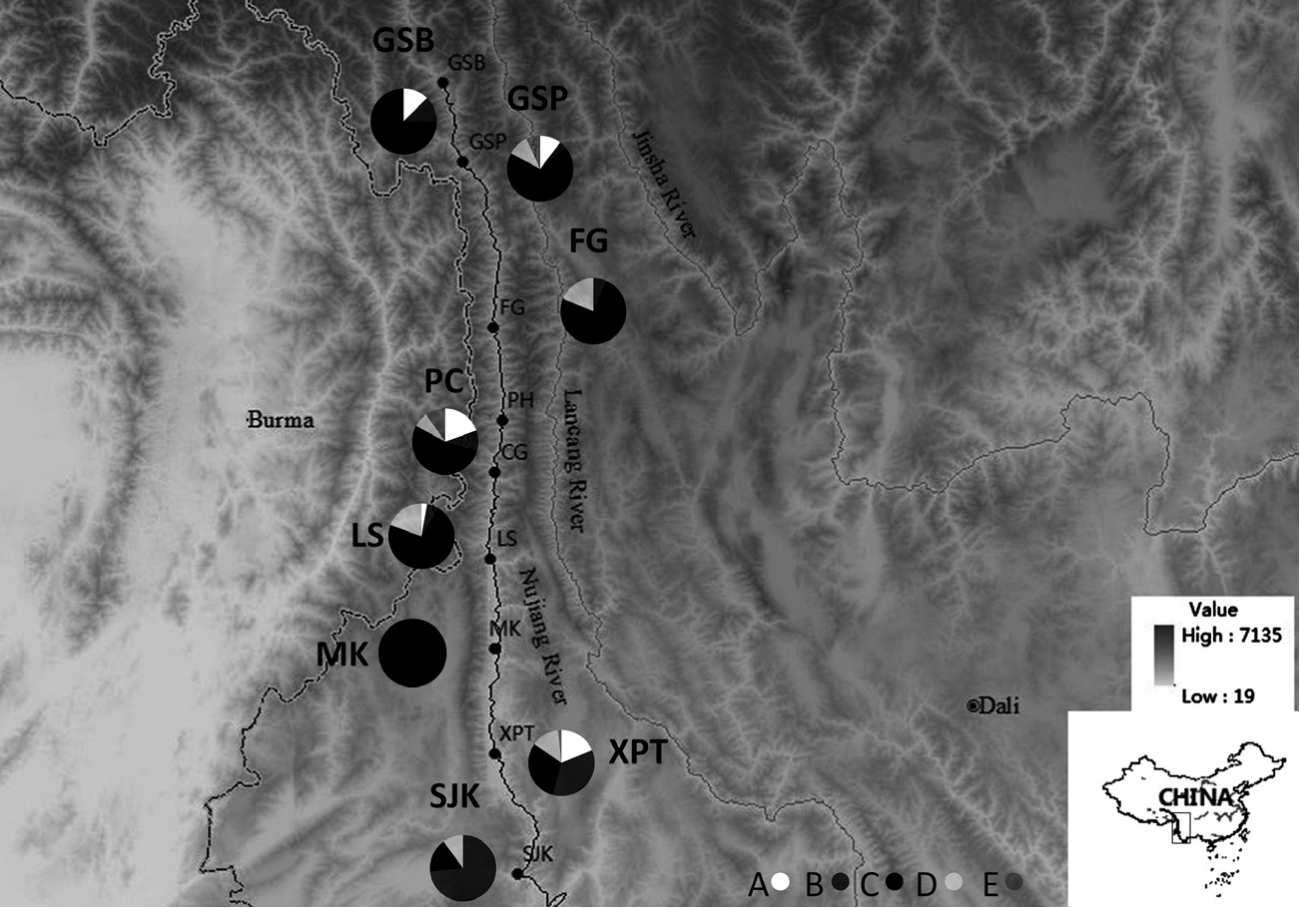
A map of the Nujiang River showing the nine sampling sites and group frequencies in each population. The information of sampling sites referred to Table1, and the five groups were defined by BAPS.

A total of 224 specimens of *S. nukiangensis* were collected from 9 localities along the Nujiang River in March and October 2012 and between May and July 2013 (Fig.2; Table1). A small piece of white muscle tissue or fin was dissected from the right body side of each specimen. All tissues used for genomic DNA extraction were preserved in 95% ethanol and deposited in the Freshwater Fish Museum at the Institute of Hydrobiology, Chinese Academy of Sciences.

**Table 1:** Descriptive statistics by sampling site for the *Schizothorax nukiangensis* in this study

Collection site	PA	Coordinates	Altitude	*N*	NH	LSH	*h* (D)	*π* (D)
Bingzhongluo	GSB	28.026/98.633	1536	8	7	2	0.964 (0.077)	0.0016 (0.0004)
Puladi	GSP	27.668/98.728	1422	29	20	14	0.966 (0.019)	0.0025 (0.0004)
Fugong	FG	26.909/98.867	1177	16	11	9	0.925 (0.050)	0.0017 (0.0003)
Pihe	PC	26.486/98.903	1036	37	20	10	0.959 (0.016)	0.0026 (0.0004)
Chenggan	PC	26.252/98.870	936	4	4	2		
Lushui	LS	25.855/98.852	824	36	18	7	0.900 (0.036)	0.0018 (0.0002)
Mangkuan	MK	25.450/98.872	747	5	4	1	0.900 (0.161)	0.0014 (0.0003)
Xiaopingtian	XPT	24.970/98.868	666	59	21	9	0.926 (0.015)	0.0021 (0.0002)
Sanjingkou	SJK	24.423/98.974	612	30	12	6	0.772 (0.075)	0.0014 (0.0003)
Nujiang River	Total			224	83	60	0.965 (0.005)	0.0024 (0.0012)

PA, population abbreviation; N, population size; Nh, number of haplotypes; LSH, location-special haplotypes; *π*, nucleotide diversity; *h*, haplotype diversity; D, standard deviation.

Total genomic DNA was extracted from muscle tissue or fin by standard salt extraction. The mitochondrial *COI* was amplified using the universal barcoding primers FishF1 and FishR1 (Ward et al. 2005). The partial mitochondrial *cytb* was amplified using the universal primers L14724 and H15915 (Xiao et al. 2001). The mitochondrial *MCR* was amplified and sequenced with the primers GEDL200 and GEDH860 (Zhao et al. 2009). The PCR contained approximately 100 ng of template DNA, 1 *μ*L of each primer (10 pmol/*μ*L), 3 *μ*L of 10× reaction buffer, 1.5 *μ*L of dNTPs (2.5 mmol/L each), and 2.0 U of Taq DNA polymerase in a total volume of 30 *μ*L. The PCR conditions for *COI* were as follows: initial denaturation at 95°C for 5 min, followed by 30 cycles of denaturation at 95°C for 1 min, annealing at 50°C for 45 sec, extension at 72°C for 45 sec, followed by a final extension at 72°C for 10 min. The PCR conditions were identical for the *cytb* gene and *MCR*, with an initial denaturation at 94°C for 3 min, followed by 30 cycles of denaturation at 94°C for 1 min, annealing at 58–64°C for 1 min, extension at 72°C for 1 min, followed by a final extension at 72°C for 5 min. The partial nuclear recombinase activating gene 2 (RAG2) sequences were obtained from a subset of samples consisting of 33 individuals with the primers RAG2-f2a and RAG2-R6a (Lovejoy and Collette 2001). The PCR amplification profile included an initial denaturation step at 94°C for 3 min, followed by 35 cycles of denaturation for 30 sec at 94°C, annealing for 30 sec at 55°C, extension for 90 sec at 72°C, and a final extension for 10 min at 72°C. The PCR products were purified by 1.0% low-melting agarose gel electrophoresis and sequenced with the same primer pair through an ABI PRISM 3700 sequencing system.

### Data analysis

The sequences were initially edited using DNASTAR multiple package (DNASTAR Inc., Madison, WI) and aligned using the CLUSTALX 2.0 program (Thompson et al. 1997). After trimming to the same length, MCD was directly used for subsequent analyses. RAG2 sequences containing more than one ambiguous site were resolved using PHASE 2.1.1 (Stephens et al. 2001; Smith et al. 2005), for which input files were prepared using SEQPHASE (Flot 2010). In our study, RAG2 sequences were only used to estimate pairwise differentiation between populations.

Genetic diversities are reflected in the nucleotide diversity (*π*) and haplotype diversity (*h*) (Nei 1987), and the standard errors for each population were calculated using DnaSP 5.10 (Librado and Rozas 2009). A network analysis was performed with Haploviewer software to estimate gene genealogies. Haploviewer software converts trees built from traditional phylogenetic methods into haplotype genealogies (Salzburger et al. 2011). The phylogeny was estimated with PhyML 3.0 (Guindon et al. 2010) using a maximum-likelihood method, and the most appropriate model of DNA substitution (HKY + I + G model), as identified by Modeltest 3.7 (Posada and Crandall 1998).

Due to the small sample size (*N* = 4), the Chenggan (CG) population was analyzed with the geographically closest population, Pihe (PH). The concatenated population was named PC and the midpoint was defined as the sample site. Pairwise genetic divergences between populations were estimated using *F*-statistics (*F*_ST_) (Weir and Cockerham 1984) with 10,000 permutations, based on the distance method. A hierarchical analysis of molecular variance (AMOVA) was used to search for significant genetic partitions among populations (Excoffier et al. 1992). Both pairwise F_ST_ comparisons and AMOVA were performed in Arlequin 3.5 (Excoffier et al. 2009). To evaluate genetic boundaries between the sampling locations studied, a spatial analysis of molecular variance (SAMOVA) was performed (Dupanloup et al. 2002). The SAMOVA algorithm was employed to search for 2 to 6 potential population units. The Bayesian method in BAPS, version 6.0 (Corander et al. 2003), was used to conduct hierarchical clustering analyses. BAPS was run using linked molecular loci with 100 replicates for each of max *K* set to 5, 10, 15, 20, 25, 30, and 35. Subsequent admixture was conducted using the number of clusters chosen in the mixture analysis with 100 iterations, 60 reference individuals, and 10 iterations for each reference individual.

Pairwise values of *F*_ST_ and *F*_ST_(1 − *F*_ST_)^−1^ (Rousset 1997) were plotted against geographic distances and the height of water among sample sites to test for IBD (Slatkin 1993). The MK population was excluded due to a small sample size and a smaller gap in the distance and water height between the LS and XPT populations. The mean of the altitude values for the PH and CG populations was used as the altitude value for the PC population. Mantel tests for IBD and water height were performed using Arlequin 3.5. Geographic distances among populations were estimated using Google Earth v.4.3.

Historical changes in effective population size were assessed using three approaches. First, Tajima's *D* (Tajima 1989) and Fu's *Fs* (Fu 1997) statistics were calculated using Arlequin 3.5 to seek evidence of demographic expansions. Second, pairwise mismatch distributions (Schneider and Excoffier 1999) were used to infer the demographic history of *S. nukiangensis* and were performed using Arlequin 3.5 and DnaSP 5.10. The previously established average substitution rate of 1.69% per million years (Zhao et al. 2009) was assumed and has been calibrated for *MCR* and the *cytb* gene in the schizothoracine fish. The average substitution rate was set at 1.50% for MCD due to the added *COI*. The expansion time was estimated from the equation *τ = 2ut* (Nei and Tajima 1981; Rogers and Harpending 1992), where *u* is the mutation rate per sequence and per generation. The value of *u* was calculated from *u = 2μk*, where *μ* is the mutation rate per nucleotide, and *k* is the number of nucleotides in the analyzed fragment. The approximate time of expansion was calculated by multiplying *t* by the generation time (4 years; Cao et al. 1981) of the schizothoracine fish. Finally, Bayesian skyline plots (BSP) (Drummond et al. 2005) were generated using BEAST 1.6.1 (Drummond and Rambaut 2007) for MCD with 100 million generations in order to explore demographic history. The HKY + I + G model and the strict clock, with a divergence rate of 1.50% per million years, were employed for MCD.

## Results

### Sequence information

All three mitochondrial fragments were successfully amplified for the 224 *S. nukiangensis* specimens. After alignment, the *COI* (675 bp) contained 13 variable sites, 7 of which were parsimony informative. The *cytb* (1063 bp) had 28 variable sites, 16 of which were parsimony informative. The *MCR* (667 bp) demonstrated 41 variable sites, 31 of which were parsimony informative. No insertions and deletions were found in any of the three gene parts. A total of 83 haplotypes were defined among the all of the MCD sequences. For RAG2, we sequenced 33 individuals from a subset of samples selected based on the MCD haplotypes. The 1126-bp fragment included five polymorphic sites, and all sequences contained five alleles. The haplotype and allele sequences were deposited in GenBank (Table S1).

### Genetic diversity and genetic structure

The number of haplotypes, location-special haplotypes and the haplotype diversity (*h*), and nucleotide diversity (*π*) values within each population and in the overall population are presented in Table1. The overall haplotype and nucleotide diversity values were 0.965 (0.005) and 0.0024 (0.0012), respectively. The highest haplotype and nucleotide diversities were discovered in the GSP and PC populations, whereas the lowest both were found in the SJK population. A total of 60 private haplotypes were detected among all sites.

The haplotype genealogy, constructed with haplotypes from different sampling localities, demonstrated little association between haplotypes and geography (Fig. S1). A star-like geography genealogy, with four main haplotypes and numerous haplotypes contained in a single individual, was obtained. Phylogenetic analyses were also conducted; however, the trees could not be resolved due to low intraspecific sequence divergence (data not shown).

**Figure 3:**
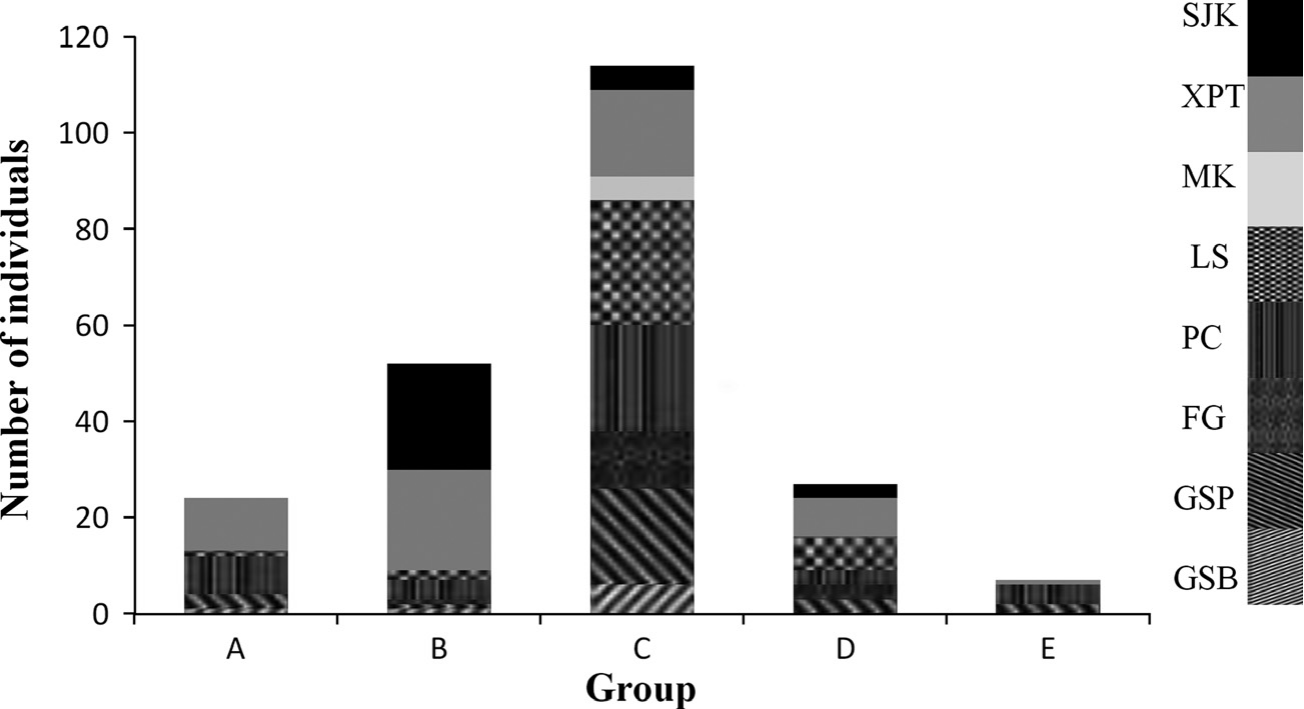
Visualization of the results of the admixture clustering that was performed in BAPS.

The nonhierarchical AMOVA revealed that most of the variation (85.14%, *P* < 0.001) occurred within sampling sites (Table S2). Global *F*_ST_ in *S. nukiangensis* was 0.149 and was significantly different from zero (*p* < 0.001). The largest mean *F*_CT_ index was found for two populations units (*F*_CT_ = 0.120, *P* < 0.05) referred to as follows: (1) XPT/SJK (D-unit); (2) the other six upstream populations (U-unit) (Table S2, Fig. S2). The estimated *F*_ST_ value in the hierarchical AMOVA based on the two units was 0.193, which was highly significant (*P* < 0.001) (Table S2). Similarly, significant genetic differentiation based on RAG2 between the two units also obtained in this study (*F*_ST_ = 0.067, *P* < 0.05) (Table S3). Clustering was performed using an admixture model in BAPS that incorporated the population of origin for each sample recovered in five groups. As shown in Figure3, most of the individuals in group B came from XPT and SJK and group C had the largest specimen number from every population. XPT contained a large number of specimens from every group, more than 70% of individuals in SJK were from group B and more than 50% of individuals in the upstream populations were derived from group C (Fig.2). Thus, Bayesian analysis showed a detectable fine-scale population structure in *S. nukiangensis*.

Pairwise comparisons of genetic differentiation (*F*_ST_) ranged from −0.019 to 0.463 and are shown in Table2. Highly significant genetic differentiations were detected between the SJK population and other populations. Furthermore, significant difference between SJK and U-unit was also found based on RAG2 (Table S3). Statistically significant differences with low values were also found when comparing the FG population to the other populations. No significant differentiations, coupled with low values, were found in the other 8 of 28 pairwise comparisons of *F*_ST_ among the sampling areas.

**Table 2:** Pairwise *F*_ST_ values and significance probability estimates

	GSB	GSP	FG	PC	LS	MK	XPT	SJK
GSB		NS	***	NS	*	NS	NS	***
GSP	−0.019		***	NS	***	NS	***	***
FG	0.214	0.123		***	***	***	***	***
PC	−0.018	0.000	0.158		***	NS	***	***
LS	0.053	0.046	0.112	0.061		NS	***	***
MK	0.077	0.029	0.219	0.031	0.023		**	***
XPT	0.096	0.100	0.151	0.109	0.115	0.175		***
SJK	0.386	0.324	0.355	0.322	0.345	0.463	0.119	

NS, nonsignificant.

Above diagonal: ^*^<0.05, ^**^<0.005, ^***^<0.0005.

**Figure 4:**
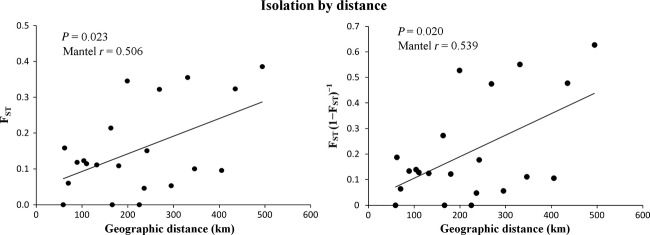
Correlation between genetic differences and geographic distances (km). p and r represent probability estimates and the correlation coefficient, respectively.

There was a statistically significant relationship between population differentiation and geographic distance (*F*_ST_: *R* = 0.5064, *P* = 0.0232; *F*_ST_(1 − *F*_ST_)^−1^: *R* = 0.5388, *P* = 0.0195) (Fig.4), but no significant relationship between population and water height (*F*_ST_: *R* = 0.2453, *P* = 0.1398; *F*_ST_(1−*F*_ST_)^−1^: *R* = 0.2780, *P* = 0.1101).

### Demographic history

**Figure 5:**
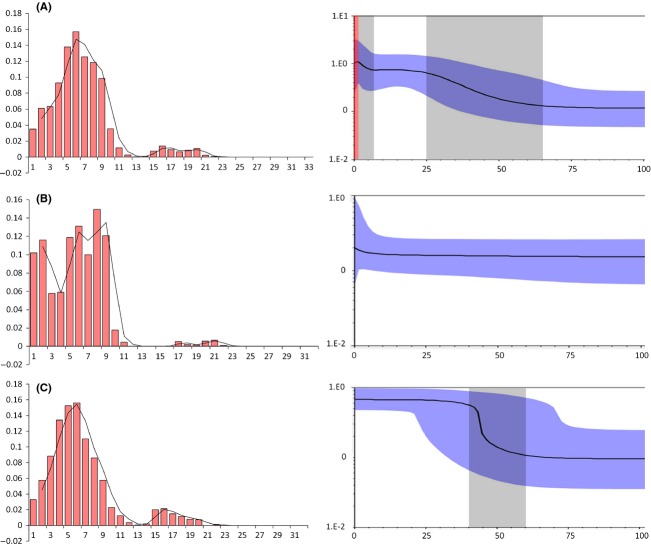
Mismatch distribution and Bayesian skyline plots (BSP) analysis. (A) All samples. (B) D-unit. (C) U-unit. Pictures in left are results of mismatch distribution. The abscissa indicates the number of pairwise differences between compared sequences. The ordinate is the frequency for each value. Histograms are the observed frequencies of pairwise divergences among sequences and the line refers to the expectation under the model of population expansion. Pictures in right are results of BSP. The abscissa shows the time in millenniums of years ago (ka). The ordinate shows the estimated effective population size. Estimates of means are joined by a solid line while the shaded range delineates the 95% HPD limits. The gray dash areas represent timescale of the expansion events, and the red dash area represents timescale of the contraction event.

The *D* and *F*_*S*_ tests implied a significant departure from neutrality for the overall population, and two population units and negative values were generated for each population by the *F*_*S*_ test (Table3). Additionally, mismatch analysis revealed approximately unimodal distribution of pairwise differences in the overall population and U-unit, but bimodal distribution in the D-unit (Fig.5). The model of sudden demographic expansion for the overall population was not rejected by the generalized least square procedure (SSD = 0.002, *P* = 0.492) or by the raggedness index of the distribution (Rag = 0.010, *P* = 0.681) (Table3). Using the evolutionary rate calibrated in this study (1.50% per Myr), the expansion times for each population and unit were estimated to be between approximately 0.069 and 0.222 Ma (Table3). For all populations, similar results were obtained with BSP, suggesting that remarkable expansion happened from 7 to 1.5 ka and moderate expansion occurred from 65 to 25 ka (Fig.5). A sharp contraction was discovered from approximately 1.5 ka to the present. For two units, by contrast, the BSP suggested that the effective population size was relatively stable in the D-unit but sharp expansion occurred in the U-unit occurred from 60 to 40 ka.

**Table 3:** Neutrality tests and mismatch distribution values for all populations

	Tajima' *D* (*P*)	Fu's *Fs* (*P*)	SSD (*P*)	Hri (*P*)	*τ*	T (Ma)
GSB	−1.13 (0.153)	−2.53 (0.047)	0.004 (0.959)	0.027 (0.961)	4.0	0.110
GSP	−1.63 (0.030)	−8.39 (0.003)	0.009 (0.238)	0.024 (0.419)	5.1	0.140
FG	−1.52 (0.056)	−3.67 (0.030)	0.010 (0.532)	0.038 (0.535)	3.9	0.107
PC	−1.08 (0.139)	−9.04 (0.004)	0.010 (0.324)	0.018 (0.418)	2.5	0.069
LS	−1.37 (0.014)	−6.50 (0.008)	0.013 (0.237)	0.045 (0.159)	5.2	0.143
MK	0.08 (0.586)	−0.13 (0.356)	0.056 (0.397)	0.150 (0.655)	4.3	0.119
XPT	−1.19 (0.106)	−4.74 (0.066)	0.009 (0.374)	0.023 (0.379)	6.1	0.169
SJK	−1.04 (0.154)	−2.66 (0.110)	0.027 (0.515)	0.040 (0.777)	8.0	0.222
U-unit	−1.86 (0.006)	−25.22 (0.000)	0.002 (0.462)	0.009 (0.795)	4.1	0.114
D-unit	−1.43 (0.047)	−8.34 (0.014)	0.011 (0.424)	0.021 (0.489)	6.6	0.183
Overall	−1.78 (0.005)	−24.94 (0.000)	0.002 (0.492)	0.010 (0.681)	5.8	0.161

*Τ*, time since expansion expressed in units of mutational time; SSD, sum of squared distribution; Hri, Harpending's raggedness index; T, estimated expansion time (Ma); *P*, the probability value.

## Discussion

Our study documents genetic structure and population demography within *S. nukiangensis* in continuous habitat. Environmental heterogeneity, limited dispersal ability and /or glacial cycles may drive genetic differentiation among populations in the fine geographic scale. Because of differential living regions and cold tolerance, *S. nukiangensis* shows differential responses to climatic fluctuations during the Pleistocene. Considering limited sample range and molecular markers in our study, our presented hypotheses were not very strong. Therefore, more sample size and molecular markers should be employed in the future to demonstrate our hypotheses.

### Population bottleneck and fine-scale population structure

In the current study, the overall haplotype and nucleotide diversities were much lower in *S. nukiangensis* compared with *S. prenanti*, *S. o'connori*, *Platypharodon extremus,* and *Gymnocypris chilianensis* (He and Chen 2009; Liang et al. 2011; Zhao et al. 2011; Su et al. 2014b); however, the results were similar to other schizothoracine fishes such as *Schizopygopsis pylzovi* and *Gymnodiptychus pachycheilus* (Qi et al. 2007; Su et al., 2014a). The high level of haplotype diversity and low nucleotide diversity in *S. nukiangensis* are indicative of a population bottleneck, followed by rapid population growth and accumulation of mutations (Grant and Bowen 1998). Population bottlenecks appear to severely impact the extent of haplotype diversity in many fish populations (Billington and Hebert 1991). Since the Quaternary period, the Tibetan Plateau has undergone several uplifts, creating substantial changes in the climate and natural environment (Li and Fang 1999) and significantly influencing the demographic history of fish species. Neutrality tests, mismatch distribution tests, and BSP analyses confirmed that a recent expansion event likely occurred in *S. nukiangensis*. Furthermore, genetic bottleneck might be also suspected in *S. nukiangensis* due to the recent population decline in response to habitat destruction and overfishing.

In contrast to cases of absence of genetic differentiation in the schizothoracine fishes (e.g., Su et al. 2014a,b), significant but moderate population genetic structure among geographic populations was detected in continuous habitat by the AMOVA, SAMOVA, BAPS, and F_ST_ analyses. The fact that nearly 73.56% of haplotypes were only detected in single locality indicates limited gene flow among populations. The most noteworthy finding was the occurrence of significant genetic differentiation between the SJK and the other populations. The significant pairwise genetic differentiation detected between the SJK population and the other populations is consistent with *Placocheilus cryptonemus* in the Nujiang River (Zhang et al. 2009) and might contribute to their specific habitat and dispersal ability. First, in contrast to turbulent water between Gongshan and Lushui, the Nujiang River is comparatively peaceful in Sanjiangkou (Liu et al. 2008). Different water environments might drive distinct ecotypes, which might produce reproductive isolation. Furthermore, higher temperatures in Sanjiangkou (Fan and He 2012) might play a role in the genetic differentiation, at least in part. A study by Li et al. (2009) found that temperature is an important variable for *Schizothorax* species richness. Because it is a cold-water fish species, it is particularly sensitive to habitat temperature. In addition, distance might also be a key cause for population differentiation (discussed below). It is also feasible that glacial cycles may have given rise to the pattern of genetic structure. Cyclic cooling potentially restricted *S. nukiangensis* to a number of confined regions, which impeded gene flow among populations. The end of the glacial periods would then be followed by a population expansion event and secondary admixture; however, they might not be a panmictic population after long-term isolation.

The other interesting finding in the current study was that Xiaopingtian might act as a mixed reservoir for *S. nukiangensis*, as evidenced by the low genetic divergence between XPT and other populations and each BAPS group containing many individuals in XPT population; however, *S. nukiangensis* rarely passed through this region. The area near Lushui might form natural barriers for fish migration and dispersal, acting as a boundary line for animal fauna in the Oriental and Tibetan Plateau areas of the Nujiang River (Chen 1998). SAMOVA, BAPS, and *F*_ST_ analyses strongly support this hypothesis.

### Complex demographic processes of *S. nukiangensis*

A gradual colonization process is expected to yield a decrease in genetic diversity in colonial populations (Ibrahim et al. 1996; Ramachandran et al. 2005; Krystufek et al. 2007). Fortunately, for the overall population, sudden population expansions can affect the genetic diversities of species and the relationships among the haplotypes. In a rapidly expanding population, more haplotypes and lineages are produced by mutations than are removed by genetic drift, which may increase the genetic diversity (Avise et al. 1984; Asmussen et al. 1987). A star-like haplotype genealogy, unimodal distribution of pairwise differences, BSP analyses, and significantly negative *F*_*S*_ values all indicate that *S. nukiangensis* has passed through recent demographic expansions.

Climatic fluctuations during the Pleistocene play a predominant role in the patterns of genetic diversity in many animals and plants, but responses to Pleistocene glacial cycles are expected to vary among species and geographic regions, in part because of differential cold tolerance (Hewitt 1996, 2004). As the Pleistocene, four or five glaciations have occurred in the Tibetan Plateau (Zheng et al. 2002). The largest glacial (LGM) development in the Tibetan Plateau happened during the middle Pleistocene (0.5 Ma), while glacial retreat has been occurring since 0.17 Ma (Zhuo et al., 1998; Zhang et al. 2000; Zheng et al. 2002). The last glaciation (LG) in the Tibetan Plateau started at approximately 0.075 Ma and continued until 0.01 Ma (Jing et al. 2004; Yi et al. 2005), after which the plateau experienced short glacial cycles with three warmer periods (Jing et al. 2004; Yi et al. 2005).

On the basis of estimated expansion times from mismatch distribution, the typical signature of the expansion events suggests that most populations underwent a rapid increase in size after the LGM, which might be related to suitable environments within their areas during warmer periods (0.17–0.075 Ma). This sharp expansion event was also detected by BSP analyses after the LG for the overall population. However, BSP also indicated that *S. nukiangensis* experienced an obvious expansion in the middle of the LG (0.075–0.01 Ma) for the overall population and U-unit population. Two reasons may account for this abnormal phenomenon. First, the habitat of *S. nukiangensis* might not have been covered by ice during the LG. *S. nukiangensis* in the current study resides at low elevation (612–1536 m), much lower than the snowline (1800–3200 m) (Shi et al. 1997; Liu et al. 1999). Second, *S. nukiangensis* dwells in an alpine environment and endured low temperatures during the ice age. In the D-unit population, climatic vacillations had little influence on *S. nukiangensis* according to the BSP result, which was likely due to the lower altitude of its living habitat and its strong suffertibility against the ice age. As for dissimilar reactions to the climatic vacillations during the LGM and LG, a major factor may explain this unusual phenomenon. Temperature in the LGM was much lower than in the LG (Zheng et al. 2002), which exceeded its cold suffertibility and might give rise to bottlenecks during the LGM. Nevertheless, *S. nukiangensis* could pass smoothly through the LG.

### Isolated by distance

Dispersal ability is severely restrained in most species (Meirmans 2012). Most animals have active locomotion, but most rarely disperse far beyond their places of birth (Greenwood 1980). The fine-scale genetic population structure seems to be a phenomenon that is generally common in poor dispersing fish species (Reusch et al. 2001; Rogers et al. 2002); thus, IBD scenarios are common phenomena in these species as well (Wang et al. 2000; Reusch et al. 2001; Taylor et al. 2001; Koskinen et al. 2002). The clear pattern of IBD in *S. nukiangensis* in the current study strongly supports the previous hypothesis and was predicted by the feeding habits, hypognathous mouth and limited capacity for dispersal of *S. nukiangensis*. Geographic distances could be a reasonable explanation for the observed geographic distribution of genetic variation between populations. *S. nukiangensis* mainly preys on algae attached to stones (Chen and Cao 2000), which would restrict its distribution range. Furthermore, limited dispersal capacity plays an important role in local adaption as well. Finally, philopatry is an essential factor that influences genetic differentiation. In contrast, water height does not present a significant barrier to gene flow in this species. Consequently, it appears that the continuous water habitat also represents a barrier for the dispersal of *S. nukiangensis*.

Unlike freshwater fish in rivers and streams with limited dispersal ability and environmental heterogeneity, pelagic fish have little opportunity for genetic diversity and isolation. The low genetic structure, on a global scale, found in *Rhincodon typus* due to its pelagic behavior provides a credible instance (Castro et al. 2007). Many other recent surveys also offer proof to explain this phenomenon (e.g., Liu et al. 2010; Papetti et al. 2012; Varela et al. 2012). While pelagic habits generally seem to prevent genetic divergence in allopatry, the river environment appears to facilitate isolation and differentiation in fish populations.

### Implications for conservation

*Schizothorax nukiangensis* is a valuable fish that is distributed along the Nujiang River. However, *S. nukiangensis* specimens taken from the Nujiang River exhibited a miniaturized body shape, greatly reflecting human disturbance, and environmental disruption. Habitat degradation and human activity likely drive the population decline in *S. nukiangensis*, but genetic factors can speed up the extinction process once a population becomes very small (Westemeier et al. 1998). Because of the low genetic diversity and recent population contraction seen in this species, it is vital that protective measures be taken immediately.

We propose several recommendations based on the current research. A nature reserve should be established with strict prohibitions on overfishing and the use of poison and explosives. In addition, special attention must be paid to the XPT and SJK populations and two units should be conserved as two separated management units (MUs) (Moritz 1994). Dam construction in the mainstream will prohibit gene flow among different subpopulations, and countermeasures should be taken to manage this. Additional analyses, using larger sample sizes and more genetic markers, should be carried out to assess the extent of isolation of the putative population of the whole Nujiang River in order to develop appropriate management measures.

## Data Accessibility

DNA sequences have been deposited in GenBank under Accession numbers KM070564–KM070812 and KP283022–KP283026. Details regarding individual samples are available in Table1 and Table S1.

## Supporting Information

**Table S1**. *Schizothorax nukiangensis* haplotype and allele frequency by population and GenBank accession number of each haplotype and allele. **Table S2**. Results of AMOVA for two grouping options of the *Schizothorax nukiangensis* estimated using F–statistics (F_ST_) based on the MCD. All *P* < 0.05. **Table S3.** Pairwise *F*_ST_ values and significance probability estimates (*P*) based on RAG2 sequences. **Figure S1**. Haplotype genealogy from Maximum Likelihood (ML) tree performed in the Haploviewer, which exhibits the relationship among haplotypes of the eight regions. **Figure S2**. Summary of results of spatial analysis of molecular variance (SAMOVA) in *Schizothorax nukiangensis* populations.
