# Supplementary material for: Genetic structure and historical demography of Schizothorax nukiangensis (Cyprinidae) in continuous habitat
Source: Ecol Evol. 2015 Feb 2;5(4):984–95. doi: 10.1002/ece3.1413 (PMC4338980; doi:10.1002/ece3.1413)

**Table S1**: *Schizothorax nukiangensis* haplotype and allele frequency by population and GenBank accession number of each haplotype and allele.

|  | GSB | GSP | FG | PH | CG | LS | MK | XPT | SJK | OVERALL | *COI* | *cytb* | *MCR* | RAG2 |
| --- | --- | --- | --- | --- | --- | --- | --- | --- | --- | --- | --- | --- | --- | --- |
| H1 |  |  |  |  | 1 | 1 |  |  |  | 2 | KM070564 | KM070647 | KM070730 |  |
| H2 |  |  |  |  | 1 |  |  |  |  | 1 | KM070565 | KM070648 | KM070731 |  |
| H3 |  |  |  |  | 1 | 4 |  |  |  | 5 | KM070566 | KM070649 | KM070732 |  |
| H4 |  |  |  |  | 1 |  |  |  |  | 1 | KM070567 | KM070650 | KM070733 |  |
| H5 |  |  | 3 |  |  | 1 |  |  |  | 4 | KM070568 | KM070651 | KM070734 |  |
| H6 |  |  | 4 |  |  |  |  | 8 | 1 | 13 | KM070569 | KM070652 | KM070735 |  |
| H7 |  |  | 1 |  |  |  |  |  |  | 1 | KM070570 | KM070653 | KM070736 |  |
| H8 |  |  | 1 |  |  |  |  |  |  | 1 | KM070571 | KM070654 | KM070737 |  |
| H9 |  |  | 1 |  |  |  |  |  |  | 1 | KM070572 | KM070655 | KM070738 |  |
| H10 |  |  | 1 |  |  |  |  |  |  | 1 | KM070573 | KM070656 | KM070739 |  |
| H11 |  |  | 1 |  |  |  |  |  |  | 1 | KM070574 | KM070657 | KM070740 |  |
| H12 |  |  | 1 |  |  |  |  |  |  | 1 | KM070575 | KM070658 | KM070741 |  |
| H13 |  |  | 1 |  |  |  |  |  |  | 1 | KM070576 | KM070659 | KM070742 |  |
| H14 |  |  | 1 |  |  |  |  |  |  | 1 | KM070577 | KM070660 | KM070743 |  |
| H15 |  |  | 1 |  |  |  |  |  |  | 1 | KM070578 | KM070661 | KM070744 |  |
| H16 |  | 1 |  |  |  |  |  |  |  | 1 | KM070579 | KM070662 | KM070745 |  |
| H17 |  | 1 |  |  |  |  |  |  |  | 1 | KM070580 | KM070663 | KM070746 |  |
| H18 |  | 1 |  |  |  |  |  |  |  | 1 | KM070581 | KM070664 | KM070747 |  |
| H19 | 2 | 4 |  | 4 |  | 10 |  | 1 |  | 21 | KM070582 | KM070665 | KM070748 |  |
| H20 |  | 3 |  |  |  |  |  |  |  | 3 | KM070583 | KM070666 | KM070749 |  |
| H21 |  | 1 |  | 2 |  |  |  |  |  | 3 | KM070584 | KM070667 | KM070750 |  |
| H22 |  | 2 |  | 1 |  |  |  |  |  | 3 | KM070585 | KM070668 | KM070751 |  |
| H23 |  | 1 |  |  |  | 1 | 1 |  |  | 3 | KM070586 | KM070669 | KM070752 |  |
| H24 |  | 1 |  |  |  |  |  |  |  | 1 | KM070587 | KM070670 | KM070753 |  |
| H25 |  | 1 |  |  |  |  |  |  |  | 1 | KM070588 | KM070671 | KM070754 |  |
| H26 |  | 1 |  |  |  |  |  |  |  | 1 | KM070589 | KM070672 | KM070755 |  |
| H27 |  | 1 |  |  |  |  |  |  |  | 1 | KM070590 | KM070673 | KM070756 |  |
| H28 |  | 1 |  |  |  |  |  |  |  | 1 | KM070591 | KM070674 | KM070757 |  |
| H29 |  | 1 |  |  |  | 1 |  | 8 | 14 | 24 | KM070592 | KM070675 | KM070758 |  |
| H30 |  |  |  |  |  | 1 |  | 3 |  | 4 | KM070593 | KM070676 | KM070759 |  |
| H31 |  |  |  |  |  | 1 |  |  |  | 1 | KM070594 | KM070677 | KM070760 |  |
| H32 |  |  |  |  |  | 5 | 2 |  |  | 7 | KM070595 | KM070678 | KM070761 |  |
| H33 |  |  |  |  |  | 2 |  |  |  | 2 | KM070596 | KM070679 | KM070762 |  |
| H34 |  |  |  | 1 |  | 1 |  | 1 |  | 3 | KM070597 | KM070680 | KM070763 |  |
| H35 | 1 |  |  | 2 |  | 1 |  | 9 | 4 | 17 | KM070598 | KM070681 | KM070764 |  |
| H36 |  |  |  |  |  | 1 |  |  |  | 1 | KM070599 | KM070682 | KM070765 |  |
| H37 |  |  |  |  |  | 2 |  |  |  | 2 | KM070600 | KM070683 | KM070766 |  |
| H38 |  |  |  |  |  | 1 |  |  |  | 1 | KM070601 | KM070684 | KM070767 |  |
| H39 |  |  |  |  |  | 1 |  |  |  | 1 | KM070602 | KM070685 | KM070768 |  |
| H40 |  |  |  |  |  |  | 1 | 1 |  | 2 | KM070603 | KM070686 | KM070769 |  |
| H41 |  |  |  |  |  |  | 1 |  |  | 1 | KM070604 | KM070687 | KM070770 |  |
| H42 |  |  |  | 1 |  |  |  |  |  | 1 | KM070605 | KM070688 | KM070771 |  |
| H43 |  |  |  | 1 |  |  |  |  |  | 1 | KM070606 | KM070689 | KM070772 |  |
| H44 |  | 2 |  | 4 |  |  |  |  |  | 6 | KM070607 | KM070690 | KM070773 |  |
| H45 |  |  |  | 2 |  |  |  |  |  | 2 | KM070608 | KM070691 | KM070774 |  |
| H46 | 1 |  |  | 1 |  |  |  |  |  | 2 | KM070609 | KM070692 | KM070775 |  |
| H47 |  |  |  |  |  |  |  |  | 1 | 1 | KM070610 | KM070693 | KM070776 |  |
| H48 |  |  |  |  |  |  |  | 3 | 2 | 5 | KM070611 | KM070694 | KM070777 |  |
| H49 |  |  |  |  |  |  |  |  | 1 | 1 | KM070612 | KM070695 | KM070778 |  |
| H50 |  |  |  |  |  |  |  |  | 1 | 1 | KM070613 | KM070696 | KM070779 |  |
| H51 |  |  |  |  |  |  |  | 2 | 2 | 4 | KM070614 | KM070697 | KM070780 |  |
| H52 |  |  |  |  |  |  |  |  | 1 | 1 | KM070615 | KM070698 | KM070781 |  |
| H53 |  |  |  |  |  |  |  | 3 | 1 | 4 | KM070616 | KM070699 | KM070782 |  |
| H54 |  |  |  |  |  |  |  |  | 1 | 1 | KM070617 | KM070700 | KM070783 |  |
| H55 |  | 3 |  |  |  |  |  |  |  | 3 | KM070618 | KM070701 | KM070784 |  |
| H56 |  |  |  | 3 |  |  |  | 7 |  | 10 | KM070619 | KM070702 | KM070785 |  |
| H57 | 1 |  |  |  |  |  |  |  |  | 1 | KM070620 | KM070703 | KM070786 |  |
| H58 |  |  |  | 1 |  |  |  |  |  | 1 | KM070621 | KM070704 | KM070787 |  |
| H59 | 1 |  |  | 1 |  |  |  |  |  | 2 | KM070622 | KM070705 | KM070788 |  |
| H60 | 1 |  |  | 2 |  |  |  |  |  | 3 | KM070623 | KM070706 | KM070789 |  |
| H61 |  |  |  | 6 |  |  |  |  |  | 6 | KM070624 | KM070707 | KM070790 |  |
| H62 |  | 1 |  |  |  |  |  |  |  | 1 | KM070625 | KM070708 | KM070791 |  |
| H63 |  |  |  | 1 |  |  |  |  |  | 1 | KM070626 | KM070709 | KM070792 |  |
| H64 |  |  |  | 1 |  |  |  |  |  | 1 | KM070627 | KM070710 | KM070793 |  |
| H65 |  |  |  | 1 |  |  |  |  |  | 1 | KM070628 | KM070711 | KM070794 |  |
| H66 |  | 1 |  |  |  |  |  |  |  | 1 | KM070629 | KM070712 | KM070795 |  |
| H67 |  |  |  |  |  | 1 |  | 2 |  | 3 | KM070630 | KM070713 | KM070796 |  |
| H68 |  |  |  |  |  |  |  |  | 1 | 1 | KM070631 | KM070714 | KM070797 |  |
| H69 |  |  |  | 1 |  |  |  |  |  | 1 | KM070632 | KM070715 | KM070798 |  |
| H70 | 1 |  |  |  |  |  |  |  |  | 1 | KM070633 | KM070716 | KM070799 |  |
| H71 |  |  |  | 1 |  |  |  |  |  | 1 | KM070634 | KM070717 | KM070800 |  |
| H72 |  | 1 |  |  |  |  |  |  |  | 1 | KM070635 | KM070718 | KM070801 |  |
| H73 |  | 1 |  |  |  |  |  |  |  | 1 | KM070636 | KM070719 | KM070802 |  |
| H74 |  |  |  |  |  | 1 |  |  |  | 1 | KM070637 | KM070720 | KM070803 |  |
| H75 |  |  |  |  |  |  |  | 3 |  | 3 | KM070638 | KM070721 | KM070804 |  |
| H76 |  |  |  |  |  |  |  | 1 |  | 1 | KM070639 | KM070722 | KM070805 |  |
| H77 |  |  |  |  |  |  |  | 1 |  | 1 | KM070640 | KM070723 | KM070806 |  |
| H78 |  |  |  |  |  |  |  | 1 |  | 1 | KM070641 | KM070724 | KM070807 |  |
| H79 |  |  |  |  |  |  |  | 1 |  | 1 | KM070642 | KM070725 | KM070808 |  |
| H80 |  |  |  |  |  |  |  | 1 |  | 1 | KM070643 | KM070726 | KM070809 |  |
| H81 |  |  |  |  |  |  |  | 1 |  | 1 | KM070644 | KM070727 | KM070810 |  |
| H82 |  |  |  |  |  |  |  | 1 |  | 1 | KM070645 | KM070728 | KM070811 |  |
| H83 |  |  |  |  |  |  |  | 1 |  | 1 | KM070646 | KM070729 | KM070812 |  |
| R1 | 3 | 1 | 4 | 2 | 2 | 6 |  | 7 | 1 | 26 |  |  |  | KP283022 |
| R2 | 1 | 2 | 2 | 3 |  |  |  | 3 | 1 | 12 |  |  |  | KP283023 |
| R3 | 1 | 3 | 2 | 5 |  |  |  | 8 | 6 | 25 |  |  |  | KP283024 |
| R4 | 1 |  |  |  |  |  |  |  |  | 1 |  |  |  | KP283025 |
| R5 |  |  |  |  |  |  |  |  | 2 | 2 |  |  |  | KP283026 |

**Table S2**: Results of AMOVA for two grouping options of the *Schizothorax nukiangensis* estimated using F–statistics (F_ST_) based on the MCD. All p < 0.05.

| Group compositions | Among groups | Among populations within groups | Within populations | F_ST_ | F_CT_ |
| --- | --- | --- | --- | --- | --- |
| One group | 14.86% | - | 85.14% | 0.149 |  |
| Two units | 12.30% | 6.99% | 80.72% | 0.193 | 0.123 |

**Table S3:** Pairwise F_ST_ values and significance probability estimates (p) based on RAG2 sequences.

|  | F_ST_ | p |
| --- | --- | --- |
| U-unit vs XPT | -0.009 | 0.454 |
| U-unit vs SJK | 0.228 | 0.005 |
| U-unit vs D-unit | 0.067 | 0.034 |
| XPT vs SJK | 0.110 | 0.098 |

**Figure S1**: Haplotype genealogy from Maximum Likelihood (ML) tree performed in the Haploviewer, which exhibits the relationship among haplotypes of the eight regions. The model of nucleotide substitution for ML was HKY + G (1.243) + I (0.926). The circles represent the haplotypes. The scale shown on the bottom right side of the figure indicates the relationship between circle sizes and the frequency of the haplotypes. Lines connecting the circles indicate a mutational step, dots in the lines represent putative mutational steps between haplotypes, and the figures in the circles represent number of individuals of the haplotype.


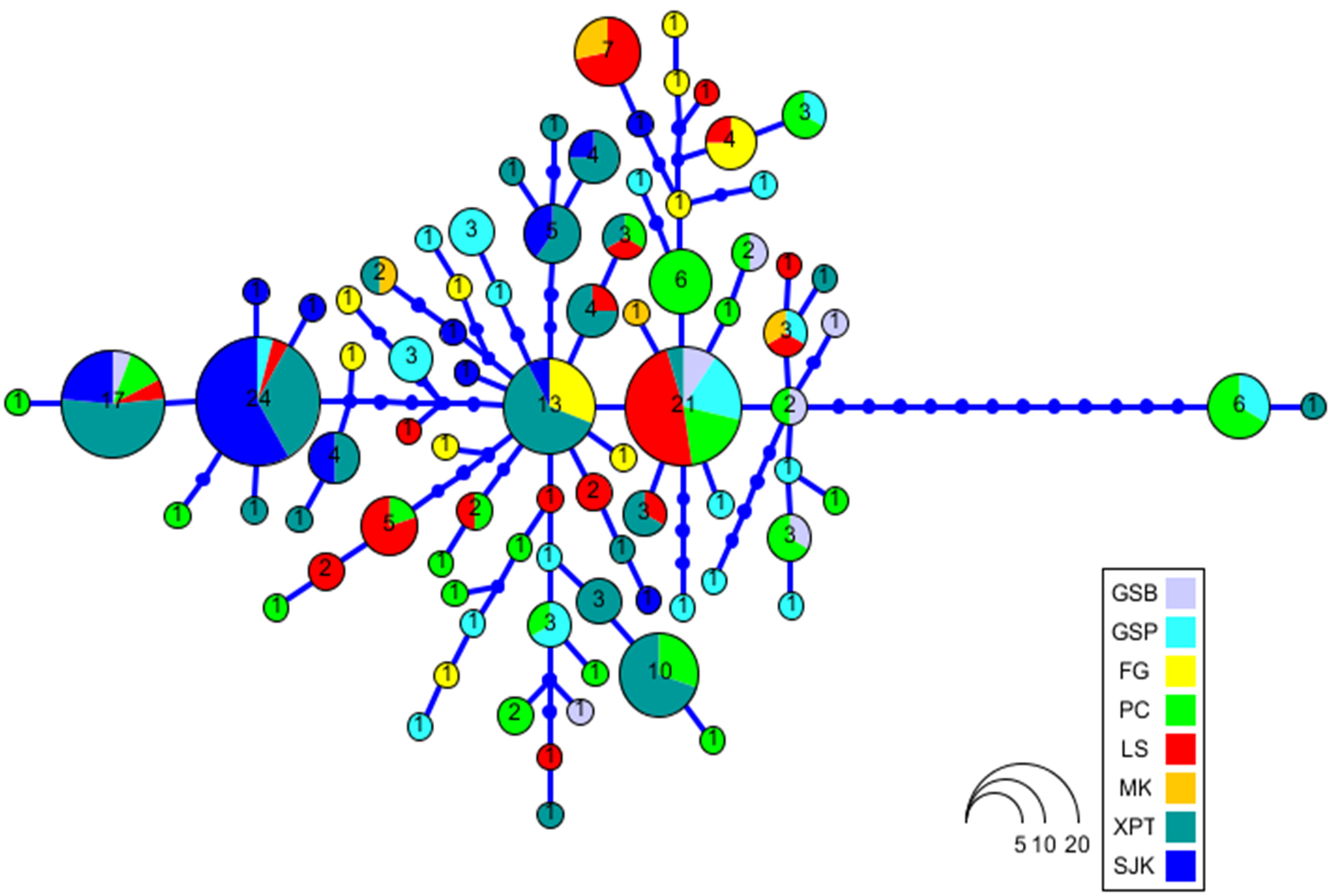


**Figure S2**: Summary of results of spatial analysis of molecular variance (SAMOVA) in *Schizothorax nukiangensis* populations.


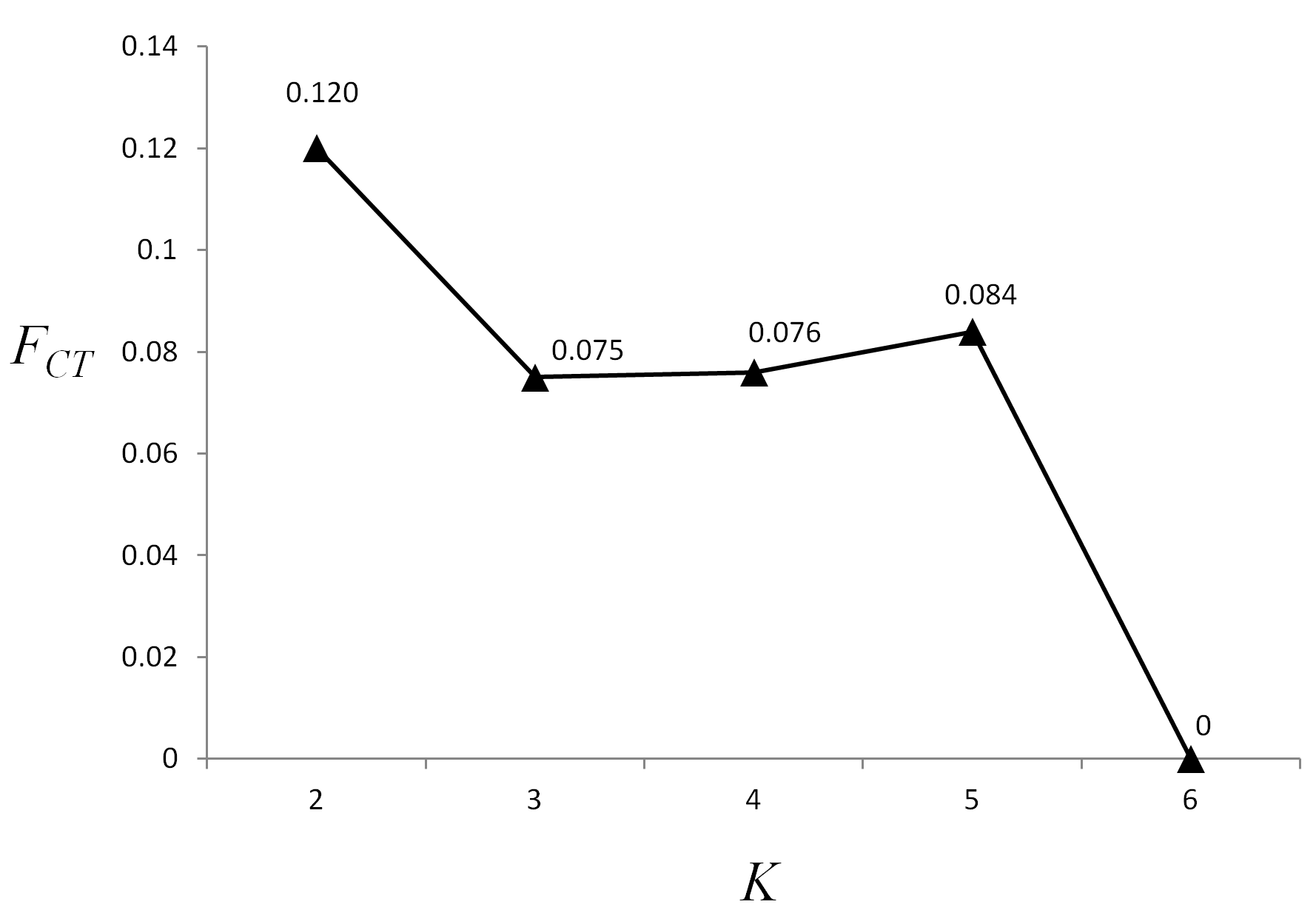

Supplement: Supplementary file 1 [file ece30005-0984-sd1.doc]
